# A novel *HNF1B* mutation p.R177Q in autosomal dominant tubulointerstitial kidney disease and maturity-onset diabetes of the young type 5: A pedigree-based case report

**DOI:** 10.1097/MD.0000000000021438

**Published:** 2020-07-31

**Authors:** Tian Tao, Yuan Yang, Zhangxue Hu

**Affiliations:** a Department of Nephrology; b Department of Medical Genetics, State Key Laboratory of Biotherapy, West China Hospital, Sichuan University, Chengdu, Sichuan, China.

**Keywords:** ADTKD, *HNF1B*, MODY5

## Abstract

**Rationale:**

Mutations in the hepatocyte nuclear factor-1-beta (*HNF1B*) gene result in a very variable presentation, including maturity onset diabetes of the young (MODY), renal cysts, renal dysplasia, and autosomal dominant tubulointerstitial kidney disease (ADTKD), which is characterized by tubular damage, renal fibrosis, and progressive renal dysfunction.

**Patient concerns:**

A 22-year-old man came to the hospital presenting with hyperglycemia, hyperuricemia and elevated serum creatinine. His urine protein was within the normal range. The ultrasound examination revealed shrunken kidneys with renal cysts. The patient's mother was diagnosed with diabetes mellitus when she was 25 years old. Her laboratory results showed elevated serum creatinine. Her ultrasonography revealed shrunken kidneys with renal cysts and hydronephrosis without kidney stones. The next-generation sequencing revealed that the proband and his mother held the same heterozygous missense mutation (c.530G>A, NM_000458, p.R177Q) in the *HNF1B* gene. Bioinformatic analyses predicted that the mutation was likely pathogenic.

**Diagnosis:**

The patient and his mother were diagnosed as ADTKD and MODY5 due to *HNF1B* mutation.

**Intervention:**

The proband was administered metformin at a dose of 500 mg/day.

**Outcomes:**

The patient had well-controlled blood glucose levels and a stable renal function at his 12-month follow-up.

**Lessons:**

We should take into account the diagnoses of ADTKD and MODY5 if patients present with early onset diabetes and multiple renal cysts or evidence of renal tubulointerstitial dysplasia, especially those with negative proteinuria results. Genetic testing helps detect the *HNF1B* gene mutations.

## 1 Introduction

Autosomal dominant tubulointerstitial kidney disease (ADTKD) is a recently defined entity characterized by autosomal dominant inheritance, bland urinary sediment with minimal blood and protein, pathological changes of tubulointerstitial fibrosis, and slowly progressive chronic kidney disease.^[1]^ A few genes with disease-causing mutations have been identified in ADTKD, including *UMOD*, *REN*, *MUC1*, *SEC61A1*, and *HNF1B. HNF1B* encodes a POU (Pit-1Oct-1/2-UNC-86) homeodomain-containing transcription factor the hepatocyte nuclear factor 1B (HNF1B). HNF1B is essential for the normal development of the kidney, liver, pancreas and other epithelial organs by regulating tissue-specific gene expression in these organs.^[2]^ ADTKD-HNF1B manifests as renal cysts, renal hypoplasia, single kidney, collecting system abnormalities, bilateral hydronephrosis, and more.^[3,4]^ The most common extrarenal manifestation is islet dysplasia and functional defect manifested as maturity-onset diabetes of the young type 5 (MODY5).^[5]^ A portion of patients present with genital tract malformations, hyperuricemia, hypomagnesemia, elevated liver enzymes, epilepsy, and autism.^[6]^

Since the first *HNF1B* mutation (p.R177X) was described in a Japanese family in 1997,^[7]^ more than 200 similar mutations have been reported, including missense/nonsense, splicing, deletions, and insertions. The majority of identified mutations are clustered in the first four exons of the gene, among which the POU domains are hot spots for mutations.^[6]^ Here, we report a Chinese family with ADTKD and MODY5. The proband and his mother were carrying a novel missense mutation (c.530G>A, NM_000458, p.R177Q) in the POU domain of the *HNF1B* gene. The detailed clinical features and pathogenesis are discussed below.

## 2 Case reports

A 22-year-old Chinese man (III:1) came to the hospital complaining of hyperglycemia revealed by a routine examination done 2 months earlier. No polyphagia, polyuria, polydipsia, and emaciation were noticed. The patient's height was 168 cm, and his weight was 70 kg, the body mass index was 24.8. His blood pressure was 138/75 mm Hg. There was no edema of the lower extremities. Laboratory tests revealed that fasting blood glucose was 130 mg/dL. The HbA1c level was 8.4%. The serum creatinine was 1.79 mg/dL. The serum uric acid was 7.92 mg/dL. The alanine aminotransferase was 69 IU/L and aspartate aminotransferase was 40 IU/L. The urinary protein creatinine ratio was 0.024 g/g. Ultrasonography showed small kidneys (left kidney: 7.5 × 4.3 × 3.1 cm, right kidney: 8.0 × 4.7 × 4.7 cm) with multiple cysts and increased cortical echogenicity. The liver and pancreas were normal. The fundus examination did not show diabetic retinopathy. The glutamic acid decarboxylase antibody (GADA), insulin autoantibody (IAA), and anti-islet cell antibody (ICA) were negative (Table 1).

**Table 1:**
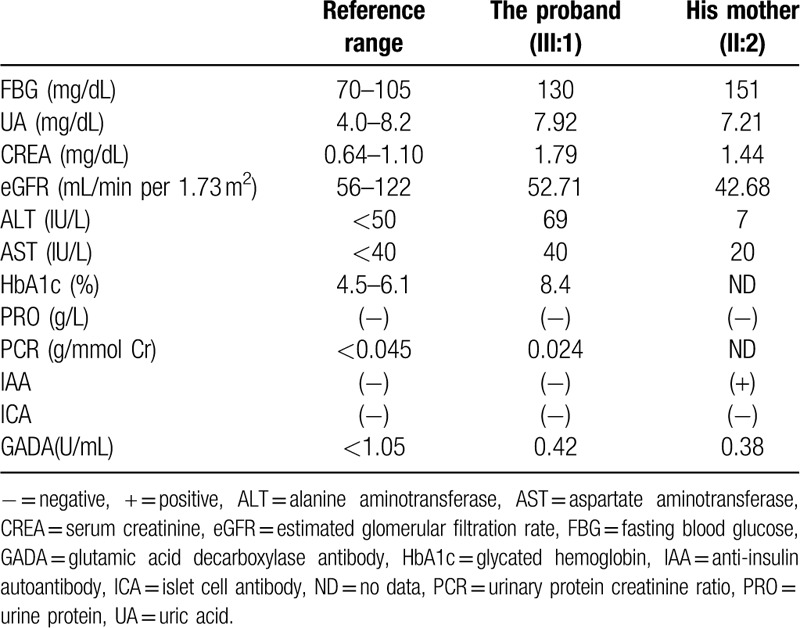
Laboratory examination.

**Figure 1:**
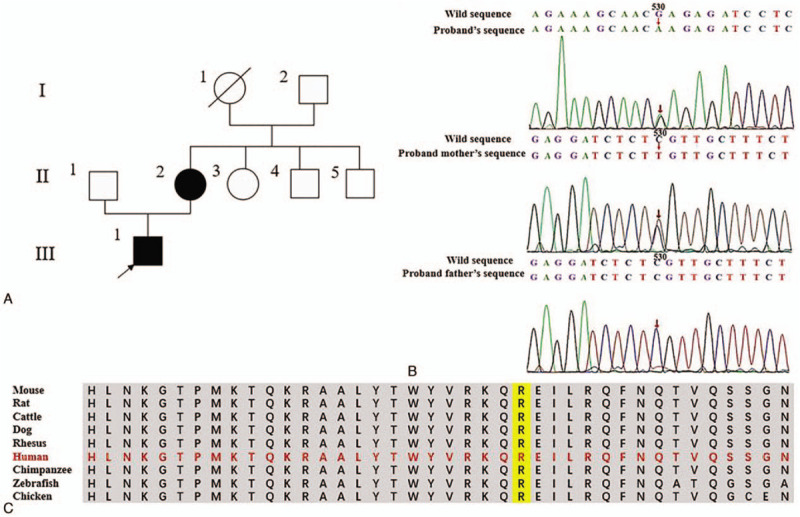
HNF1B sequencing and genetic analysis of the patient‘s family. (A) Showed the family pedigree. The proband‘s uncle (II:5) was diagnosed as diabetes mellitus. Other clinical data were not available. He did not take the gene sequencing. The proband's grandmother (I:1) had Alzheimer's disease. She is dead and no clinical data were available. (B) showed the *HNF1B* mutation c.530G>A (p.R177Q) by Sanger sequencing, which was held by the proband (III:1) and his mother (II:2), not by his father (II:1). (C) showed that the amino acid residue R177 in the POU domain of the *HNF1B* gene was highly conserved among species.

His mother (II:2), a 47-year-old woman, was diagnosed with diabetes mellitus when she was 25 years old, after which she was put on insulin therapy. Laboratory tests showed that fasting blood glucose was 151 mg/dL and the serum creatinine level was 1.44 mg/dL. The serum uric acid was 7.21 mg/dL. The alanine aminotransferase was 7 IU/L and aspartate aminotransferase was 20 IU/L. Her urine tested negative for protein. The ultrasonography showed small kidneys (left kidney: 8.1 × 3.9 × 4.7 cm, right kidney: 7.9 × 4.0 × 3.2 cm) with multiple cysts and hyperechogenic cortical ultrasonography. Besides, bilateral hydronephrosis without kidney stones was revealed. Her GADA and ICA were negative, IAA was positive (Table 1). The health of the proband's father (II:1) was sound. The proband‘s uncle (II:5) was diagnosed as diabetes mellitus (Fig. 1A).

After written informed consent was obtained, next-generation sequencing (NGS) was performed. A missense mutation, c.530G>A (p.R177Q) in the *HNF1B* (reference sequence GenBank accession no. NM_000458) was identified in the proband and his mother. The sanger sequence confirmed the mutation (Fig. 1B). The proband's father did not carry the mutation. Other family members did not undergo gene sequencing. The mutation was located in the POU-specific domain of HNF1B, and the amino acid residue R177 was highly conserved among species according to the Clustal Omega program designed by the European institute for biological information (EBI) (https://www.ebi.ac.uk/Tools/msa/clustalo/) (Fig. 1C). The mutation was absent from the Human Genetic Mutation Database (HGMD) and the 1000 Genomes Project Data. PolyPhen2 analysis predicted that the p.R177Q mutation is probably damaging, with scores of 1.00 in HumDiv and HumVar models. The MutationTaster2 predicted the mutation to be pathogenic. The p.R177Q mutation in population frequencies is 8.237e-06 from the Exome Aggregation Consortium. According to the American College of Medical Genetics and Genomics guidelines and standards, the c.530G>A (p.R177Q) was estimated as likely pathogenic.

The proband and his mother were diagnosed with *HNF1B*-associated ADTKD and MODY5. The proband received metformin at a dose of 500 mg/day to lower the blood glucose. He had well-controlled blood glucose levels and a stable renal function during the 12-month follow-up.

## 3 Discussion

In humans, the *HNF1B* gene is located on chromosome 17q12. The phenotypic expression of *HNF1B* is seen in the kidney, liver, pancreas, urinogenital tract, gut, and bile ducts. The encoded transcription factor HNF1B contains 557 amino acids with three distinct domains: the dimerization domain, the DNA-binding domain and the transactivation domain.^[6]^ HNF1B binds to DNA to regulate tissue-specific gene expression in different organs.

A total number of 286 *HNF1B* mutations have been documented in the HGMD (accessed on the May 12, 2020), with approximately 50% of patients having either a missense or nonsense mutation and others having splice site mutations, small insertions/deletions or gross deletions. The highly conserved DNA-binding domain of HNF1B is an essential area for transcription regulation, which is also a hot spot for mutations.^[8]^

The phenotype of *HNF1B* mutation carriers is extremely variable with autosomal dominant traits. Half of the patients do not have any family history because of spontaneous mutations. The kidney is the most commonly affected organ, with manifestations that include tubulointerstitial disease with cysts, hypoplastic glomerulocystic kidney disease, unilateral multicystic dysplasia, hypodysplasia, unilateral agenesis and hydronephrosis. Electrolyte abnormalities can also occur. About 5% to 31% of congenital abnormalities of the kidney and urinary tract are associated with *HNF1B* mutations. Clinically HNF1B nephropathy manifests as chronic renal failure with little or no proteinuria (<1 g/day) and no hematuria. HNF1B nephropathy has a slow-progressive phenotype in childhood except for very early onset cases.^[9]^ About 3% to 15% of these patients develop end-stage renal disease. The detailed pathogenic mechanism has not yet been elucidated. In the stage of nephrogenesis, *HNF1B* gene deficiency causes abnormal ureteric bud branching and deformed S-shaped bodies generating, which finally differentiate into the Bowman‘s capsule and tubules.^[3]^ Inactivation of *HNF1B* in the metanephric mesenchyme leads to the formation of aberrant nephrons.^[10]^*HNF-1B* gene mutations could directly downregulate the transcription of genes like Pkhd1, Pkd2, Umod, Glis2, and Kif12, which result in the production of kidney cysts.^[3,11,12]^*HNF1B* gene deficiency also induces renal fibrosis through epithelial-mesenchymal transition and aberrant TGF-b signaling.^[2]^ All the above contribute to the onset of HNF1B-associated ADTKD.

The pancreas is the most commonly affected extra-renal organ. The patients may present with pancreatic hypoplasia, agenesis (75%) and insulin-dependent diabetes mellitus (∼45%). HNF1B-associated diabetes mellitus represents ∼1% to 6% of MODY cases in the United Kingdom. Diabetes typically develops during adolescence or early adulthood and the mean age at diagnosis of diabetes is 26 years. HNF1B plays an important role in the early development of the pancreas, participating in the proliferation of pancreatic multipotent progenitor cells, pancreatic duct and islet cells.^[13]^*HNF1B* gene deficiency can significantly reduce the number of pancreas-associated endocrine cells and insulin secretion.^[14]^ Besides, the mutations in the HNF1B DNA-binding domain seems to limit the ability to increase the transcription of glucose transporter 2, which could sense external glucose and transport it into pancreatic endocrine cell.^[14]^

Here, we presented a family with ADTKD and MODY5. A novel *HNF1B* mutation at the guanine-recognizing residue R177(c.530G>A) was identified in the proband and his mother. The family study revealed the autosomal dominant trait. Interestingly, the first reported *HNF1B* mutation (c.529C>T, p.R177X) sat in the same locus.^[7]^ The R177X mutation generates a protein of 176 amino acids with the N-dimerization and POU domains. The truncated protein does not stimulate transcription and seems to be a loss-of-function mutation. As a missense mutation, the R177Q mutation seems to be less pathogenic than the R177X mutation. However, the R177Q mutation also caused ADTKD and MODY5, which suggest the importance of POU-containing domains in *HNF1B*. The proband's mother maintained mildly increased serum creatinine and bland urinary sediments 20 years after the onset of MODY, suggesting that the R177Q mutation may lead to a slow-progressive kidney phenotype.

In conclusion, we identified a novel *HNF1B* mutation (c.530G>A, NM_000458, p.R177Q) in a Chinese family with typical phenotypes of ADTKD and MODY5. This case suggests that a missense mutation in POU-containing domains in *HNF1B* may be pathogenic. The presentation of the *HNF1B* mutations is highly variable. Accurate genetic diagnosis is important. The NGS could help in detecting the gene mutations.

## Acknowledgments

The authors are grateful to all the study participants. We thank Enago Academy (www.enago.cn) for their help in language editing.

## Author contributions

**Conceptualization:** Tian Tao.

**Data curation:** Tian Tao, Zhangxue Hu.

**Formal analysis:** Tian Tao, Yuan Yang, Zhangxue Hu.

**Methodology:** Yuan Yang, Zhangxue Hu.

**Project administration:** Tian Tao, Zhangxue Hu.

**Software:** Tian Tao.

**Supervision:** Zhangxue Hu.

**Writing – original draft:** Tian Tao, Zhangxue Hu.

**Writing – review & editing:** Tian Tao, Zhangxue Hu.
